# Mortality Predictive Value of APACHE II and SOFA Scores in COVID-19 Patients in the Intensive Care Unit

**DOI:** 10.1155/2022/5129314

**Published:** 2022-03-28

**Authors:** Mohammad Taghi Beigmohammadi, Laya Amoozadeh, Forough Rezaei Motlagh, Mojgan Rahimi, Maziar Maghsoudloo, Behzad Jafarnejad, Babak Eslami, Mohammad Reza Salehi, Kazem Zendehdel

**Affiliations:** ^1^Department of Anesthesiology and Intensive Care, Imam Khomeini Hospital Complex, Tehran University of Medical Sciences, Tehran, Iran; ^2^Research Center for War-Affected People, Tehran University of Medical Sciences, Tehran, Iran; ^3^Department of Infectious Diseases, Imam Khomeini Hospital Complex, Tehran University of Medical Sciences, Tehran, Iran; ^4^Cancer Research Center, Cancer Institute of Iran, Tehran University of Medical Sciences, Tehran, Iran

## Abstract

**Background:**

COVID-19 pandemic has become a global dilemma since December 2019. Are the standard scores, such as acute physiology and chronic health evaluation (APACHE II) and sequential organ failure assessment (SOFA) score, accurate for predicting the mortality rate of COVID-19 or the need for new scores? We aimed to evaluate the mortality predictive value of APACHE II and SOFA scores in critically ill COVID-19 patients.

**Methods:**

In a cohort study, we enrolled 204 confirmed COVID-19 patients admitted to the intensive care units at the Imam Khomeini hospital complex. APACHE II on the first day and daily SOFA scoring were performed. The primary outcome was the mortality rate in the nonsurvived and survived groups, and the secondary outcome was organ dysfunction. Two groups of survived and nonsurvived patients were compared by the chi-square test for categorical variables and an independent sample *t*-test for continuous variables. We used logistic regression models to estimate the mortality risk of high APACHE II and SOFA scores.

**Result:**

Among 204 severe COVID-19 patients, 114 patients (55.9%) expired and 169 patients (82.8%) had at least one comorbidity that 103 (60.9%) of them did not survive (*P*=0.002). Invasive mechanical ventilation and its duration were significantly different between survived and nonsurvived groups (*P* ≤ 0.001 and *P*=0.002, respectively). Mean APACHE II and mean SOFA scores were significantly higher in the nonsurvived than in the survived group (14.4 ± 5.7 vs. 9.5 ± 5.1, *P* ≤ 0.001, 7.3 ± 3.1 vs. 3.1 ± 1.1, *P* ≤ 0.001, respectively). The area under the curve was 89.5% for SOFA and 73% for the APACHE II score. Respiratory diseases and malignancy were risk factors for the mortality rate (*P*=0.004 and *P*=0.007, respectively) against diabetes and hypertension.

**Conclusion:**

The daily SOFA was a better mortality predictor than the APACHE II in critically ill COVID-19 patients. But they could not predict death with high accuracy. We need new scoring with consideration of the prognostic factors and daily evaluation of changes in clinical conditions.

## 1. Introduction

In December 2019, severe acute respiratory syndrome coronavirus 2 (SARS-CoV-2) spread throughout China and then over the world and caused the COVID-19 pandemic [1]. According to the World Health Organization update (15 December 2020), about one year since the start of the pandemic, 70 million accumulative cases and 1.6 million deaths have been reported [2]. The mortality range in critically ill patients with COVID-19 is as wide as 11–61% [3]. There are some predictors for disease severity include old age [4], comorbidities, such as cardiovascular disease, chronic kidney disease, chronic lung disease, and diabetes mellitus [5], and laboratory tests and markers such as white blood cell (WBC) count [6], D-dimer [7] and lactate dehydrogenase (LDH) [8]. APACHE II (acute physiology and chronic health evaluation) and SOFA (sequential organ failure assessment) scores are the most well-known scoring systems, used for many years evaluating non-COVID-19 critical patients and predicting their mortality. The APACHE II score is a system for classifying disease severity in acutely ill patients, based on age, past medical history, and some physiologic measurements that can facilitate the evaluation of treatment, the patient's prognosis, and making decisions for the allocation of hospital resources [9]. The SOFA score assesses organ dysfunction, morbidity, and mortality in the ICU according to the level of function in six organ systems: respiratory, circulatory, renal, hematology, hepatic, and central nervous system [10].

However, despite a few studies [11, 12], there is no specific and accurate scoring system for the mortality prediction of COVID-19 patients yet. They focused on the determination of the risk of disease and treatment strategies. One way is to utilize familiar and available scoring tools, which have been applied for many years in critically ill patients, to evaluate COVID-19 patients' outcomes. Nevertheless, the present scoring tools may not provide an accurate assessment of COVID-19 patients and need more studies for them. [13] A sound scoring system helps clinicians screen patients, select effective strategies rapidly, maximize treatment success, and minimize the wasting of limited resources during the COVID-19 pandemic. To design an assured and accurate score, we need many studies about various aspects of COVID-19. Accordingly, we aimed to evaluate the predictive value of APACHE II and SOFA scores in predicting the prognosis and mortality of COVID-19 patients.

## 2. Material and Methods

### 2.1. Study Population and Setting

The study was approved by the university ethics committee (IR.TUMS.VCR.REC. 1399.192), and written informed consent was obtained from all subjects, and in the situation that they were not eligible for informed consent, we obtained it from their relatives. A cohort study was conducted to evaluate the predictive value of APACHE II and SOFA scores in 259 patients admitted to intensive care units (ICUs) of the general tertiary Imam Khomeini hospital complex (include Cancer institute, Imam, and Valiasr hospitals), Tehran University of Medical Science, Tehran, Iran. A total of 204 participants were investigated. The participants included patients with laboratory-confirmed COVID-19 with positive SARS COV 2 RT-PCR from nasopharyngeal swab or respiratory secretions, from both sexes, and age>16 years. We searched for all adult patients who had been diagnosed with severe/critical COVID-19. COVID-19 patients who were transferred to the ICU for reasons other than severe COVID-19 were excluded. Severe COVID-19 criteria: shortness of breath, with RR ≥ 30 times/min, oxygen saturation ≤93%, oxygenation index ≤300 mmHg, and chest radiographic images showed more than 50% of the affected tissue within 24–48 h. [14].

### 2.2. Data Collections and Outcomes

Demographic data, comorbidities and chronic medical conditions, vital signs, and laboratory values (as based value) were registered on the ICU admission day. APACHE II and SOFA scoring were performed and recorded in the first 24 hours and day to day for SOFA score. The primary outcome was mortality in the ICU, hospital, or discharge. All patients were followed up to determine this outcome, and the relevant information was recorded, and the secondary outcome was organ dysfunction. Other data such as ICU and hospital length of stay and the need for different ventilation support types were considered factors that may affect patients' outcomes. Laboratory routine tests for COVID-19 management were run, and results were collected. The worse values and vital signs associated with the worse medical conditions in the first 24 hours were used to calculate the APACHE II score and approximate ICU mortality rates. For SOFA score, the first day and mean of daily SOFA scores during ICU admission were calculated. We recorded adverse effects on the kidney (acute kidney injury, oliguria, anuria, and need for dialysis), cardiac (new abnormalities in electrocardiography, rise of serum levels of troponin I, decrease of ejection fraction under 40%, need to vasopressor/inotrope), liver (an increase of liver enzymes and bilirubin, abnormal INR), hematologic (leukocytosis, lymphopenia, neutrophilia, anemia, platelet count <100000 *μ*/ml), gastrointestinal (bleeding, diarrhea, melena), neurologic (loss of consciousness, seizure, lethargy, delirium, paresthesia/plegia), and respiratory (acute respiratory distress syndrome, hemoptysis, pneumothorax, emphysema, effusion) as the adverse organic outcomes due to COVID-19 disease. Acute kidney injury (AKI) was identified based on Acute Kidney Injury Network (AKIN) criteria [15]. ARDS was defined according to the Berlin definition [16].

### 2.3. Statistical Analysis

Two groups of survived and nonsurvived patients were compared by the chi-square or Fisher's exact test for categorical variables. An independent sample *t*-test was applied to study the different continuous variables between the two groups. Values are shown as mean ± SD or as number and percentage for continuous variables and categorical variables, respectively. We used the receiver operating characteristic (ROC) curve to verify the best cut-off points for APACHE II and SOFA scores. The area under the curve (AUC) for APACHE II score 13 and SOFA score 5 were used to categorize the patients and include them in the model. We grouped important comorbidities, including diabetes mellitus, hypertension, malignancy, respiratory disease, kidney disease, gastrointestinal diseases, and hepatobiliary disorders and created a variable to adjust for the comorbidities confounding effect. We used logistic regression models to estimate the risk of high APACHE II and SOFA scores on death due to COVID-19 among patients admitted to ICUs. We also adjusted for comorbidities and sex in the model. In this study, *P* value of less than 0.05 was considered statically significant. Statistical analyses were carried out by SPSS version 16.0 for Windows.

## 3. Results

In this study, 259 ICU patients were evaluated, and finally, 204 patients (130 (63.7%) men and 74 (36.3%) women) with confirmed COVID-19 have been analyzed. Among these, 90 (44.1%) patients recovered, and 114 (55.9%) expired. The mean age in the nonsurvived and survived group was not significantly different, the same as sex. In noninvasive ventilation supports, the difference between survived and the nonsurvived group was not remarkable. However, all 9 cases that only received bilevel positive airway pressure (BiPAP) or continuous positive airway pressure (CPAP) belonged to the nonsurvived group (Table 1).

Complications, except GI complications, in nonsurvived patients were higher than survived ones (Table 3). The bacterial positive respiratory tract culture (14.2%) was significantly higher in the nonsurvived group (72.4%, *P* ≤ 0.001). Acinetobacter spp. (52.4%) and Klebsiella spp. (87.5%) were the most microorganisms in the survived and nonsurvived groups, respectively. Blood cultures were positive for 32 (15.7%) cases, with most *Staphylococcus aureus* in both groups (10 vs. 22). However, the rate of positive blood culture was not significantly different between the two groups (*P*=0.124).

Among laboratory findings, mean values of every day checked tests: WBC, neutrophil and lymphocyte count, platelet count, urea, creatinine, direct bilirubin, albumin, calcium level, pH, and base excess, were significantly different between the two groups (*P* ≤ 0.001) (Table 4).

## 4. Discussion

Since the COVID-19 pandemic spread across the world, health care systems have faced new challenges in predicting morbidity and mortality in patients with COVID-19. In this epidemiologic and clinical observational study, the mortality rate for ICU based on APACHE II score and mean SOFA value of admission days was 21.5% and 7.3%, respectively. Nevertheless, the true mortality rate (55.9%) was very different from the mortality prediction of APACHE II and SOFA scores. Nonetheless, both scores showed an increase in the mortality rate at a higher score value (*P* ≤ 0.001). With a cut-off point of 13 for APACHE II and 5 for SOFA score, the mean daily SOFA score had a better predictive performance (*P* ≤ 0.001). Against, Zou et al. [13] showed APACHE II was a better predictor for hospital mortality than SOFA with a cut-off point of 17 and 3, respectively. This discrepancy may be due to differences in the SOFA score assessment. We recorded SOFA daily, but Zou et al. [13] just included first-day scoring. The ascending trend in SOFA score significantly leads to higher mortality in ICU COVID-19 patients. [17] On the other hand, daily assessment of the SOFA score is vital because of on-time recognition of the necessity of treatment intervention, as for fast changes in the clinical situation and consequently the prognosis. Liu et al. [3] compared the function of SOFA and qSOFA in predicting mortality in 140 COVID-19 patients. SOFA score with a cut-off point of ≥3 and sensitivity of 90%, and specificity of 83.18% had better performance than qSOFA. On the contrary, in a study of 675 COVID-19 patients with respiratory failure, the SOFA score was not an accurate mortality predictor before intubation. [18].

Cheng et al. [14] showed that the APACHE II score was overall a better predictor of severity of disease and mortality in comparison with MuLBSTA (multi lobular infiltration, hypo-lymphocytosis, bacterial coinfection, smoking history, hypertension, and age) and CURB-65 (Confusion, Urea, Respiratory rate, Blood pressure, Age≥ 65) in COVID-19 patients. The same as our study, Stephens et al. [19] declared the APACHE II score underestimated mortality risk in COVID-19 patients as well as the severity of the disease. In critically ill patients, the mortality rate of COVID-19 was higher than severe acute respiratory syndrome coronavirus (SARS-CoV) and Middle Eastern respiratory syndrome coronavirus (MERS-CoV). [14] In our study, almost half of the critically ill patients died. Some of the reasons include, a large number of COVID-19 admissions in a short time led to a reduction in quality of care. We educated our staff for improvement of care. Second, COVID-19 disease was unknown at the start of the epidemic as an emerging infectious disease. We had a delay in the run of treatment protocols and coordination of policies. This particular situation needs an accurate clinical predictive tool for appropriate and on-time decisions.

In our study, the mean SOFA was a better predictor score than the APACHE II score, and its cut of value was lower than mortality prediction. Some reasons can explain it: First, the APACHE II score was assessed only on the first day of ICU admission. A more variable value of the APACHE II score was standard on admission day, and its value was zero. Second, a small number of patients had organ failure or underwent surgery, which had a high score on the APACHE II calculation. For these reasons, the APACHE II score could not show the patient's condition commensurate with the severity of the disease.

In contrast, we assessed the SOFA score daily. Oxygenation and ventilation support are the most critical variables in calculating the SOFA score. Many critically ill patients need advanced ventilation support during their stay in the ICU. Also, kidney injury is another crucial variable in the SOFA score. In our study, half of the patients suffered kidney complications during admission. Accordingly, assessing the daily SOFA score could evaluate better the patient's condition.

Old age was presented to be a major factor for predicting mortality and severity in COVID-19 patients, and male sex was associated with higher mortality. [20, 21] In our study and other [13, 22] studies, the male sex was not a risk factor for COVID-19 patients' mortality significantly.

We found a significant relationship between comorbidities and morbidity in COVID-19 patients like many other studies [13, 23, 24], and frequent high comorbidities were the same as in most of them. It could show the relationship between the damages that hypertension, cardiovascular disease, or diabetes cause in the endothelium of essential organs like the heart, lung, and kidney. [23, 24] Therefore, they are prone to early and prolonged hypoxia. On the other side, endothelial damage runs unfavorable preconditions result in thrombosis formation which is a cause of adverse outcomes in COVID-19 patients. [23, 24] Figliozzi's [21] meta-analysis showed an increase in the combined adverse outcome in patients with COVID-19 who had a positive history of DM, HTN, cardiovascular disease, cerebrovascular disease, and cancer, as well as in Tian's et al. [25] study. Like us, in another study, hypertension and diabetes were the most common factors in patients admitted with COVID-19 [26]. They also showed that in the nonsurvived group, diabetic patients were more likely to receive mechanical ventilation or intensive care. Interestingly, they found that the need for ICU care and mechanical ventilation decreased in hypertensive patients. Like them [26], we found that hypertension is not a risk factor for morbidity and mortality in COVID-19 patients. This result may be due to the protective effect of angiotensin receptor blockers which almost all our hypertensive patients received to control their hypertension. [27, 28].

Organ complications are risk factors for mortality [13, 29–31]. The most complications include ARDS, AKI, acute cardiac injury, acute liver injury, and shock. Of course, Zhou [29] showed that AKI and coinfection were the main complications in prolonged hospitalization.

In Atieh et al. [32] meta-analysis of 19 articles, significant lab findings with prognostic value included leukocytosis, lymphopenia, neutrophilia, thrombocytopenia, increased D-dimer, decreased fibrinogen, increased CRP and procalcitonin, increased creatinine, total bilirubin, ALT, AST, LDH, and decreased albumin. Our findings repeated these results except for AST and procalcitonin.

Our study has some limitations. It is a retrospective study, and the data were collected in a critical condition. Probably, there is a certain degree of clinical data deficiency. Our hospital is a tertiary mega hospital and referral center that includes three hospitals with multiple intensive units that may be the reason for our study's high mortality. We are confused about COVID-19 characteristics yet. Therefore, we need further studies to understand COVID-19 disease better.

## 5. Conclusion

The mean daily SOFA score was a better mortality predictor tool than the APACHE II score in critically ill COVID-19 patients. Since the actual mortality is significantly more than the prediction of these tools, the need to validate scoring for the prediction of mortality in COVID-19 is necessary. Alongside, many other factors, such as comorbidities, complications, and lab findings, had an essential role in the prognosis of these patients. It seems that for the innovation of a more accurate scoring system, two points should be considered, these prognostic factors in COVID-19 and the daily evaluation of changes in clinical conditions.

## Figures and Tables

**Figure 1:**
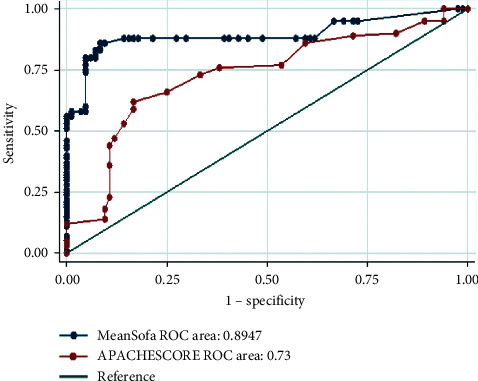
Comparison of the ROC for mean SOFA and APACHE II score of patients hospitalized for COVID-19 at ICU. APACHE II: acute physiology and chronic health evaluation, SOFA: sequential organ failure assessment.

**Table 1:** Baseline characteristics of survived and nonsurvived patients.

Variable	Survived	Nonsurvived	*P* value	95% CI	
				Lower	Upper
Sex, *n* (%)	90 (44.1)	114 (55.9)		—	—
Male	54 (60)	76 (66.7)	0.379		
Female	36 (40)	38 (43.3)			
Age (years), mean ± SD	61.4 ± 14.7	63.3 ± 12.3		−5.657	1.954
<60, *n* (%)	41 (45.5)	46 (40.3)	0.347		
≥60, *n* (%)	49 (54.5)	68 (59.7)	0.479		
BMI, mean ± SD	27.9 ± 3.9	27.4 ± 5.1		−1.721	1.043
<30, *n* (%)	62 (81.6)	78 (78.8)	0.629		
≥30, *n* (%)	14 (18.4)	21 (21.2)	0.397		
Comorbidities, *n* (%)				—	—
Any	66 (73.3)	103 (91.2)	0.002		
Diabetes	45 (39.5)	34 (37.8)	0.885		
Malignancy	19 (16.7)	6 (6.7)	0.033		
Respiratory	16 (14)	3 (3.3)	0.013		
Hypertension	38 (33.3)	42 (46.7)	0.061		
Heart	31 (27.2)	26 (28.9)	0.875		
GI	0	1	NA		
Hepatic	4	0	NA		
Kidney	10 (8.8)	3 (3.3)	0.152		
Endocrine	9 (7.9)	7 (7.8)	0.894		
Others	19 (21.1)	21 (18.4)	0.287		
APACHE II score, mean ± SD	9.5 ± 5.1	14.4 ± 5.7	≤0.001	−6.486	−3.406
APACHE II mortality prediction, %, mean ± SD	13.1 ± 10.1	21.5 ± 12.4	≤0.001	−11.520	−5.066
SOFA (first day), mean ± SD	3.2 ± 1.1	5.6 ± 3.4	≤0.001	−3.121	−1.619
Mean SOFA, mean ± SD	3.1 ± 1.1	7.3 ± 3.1	≤0.001	−4.940	−3.578
Length of stay, mean ± SD					
ICU, day	7.9 ± 46	8.1 ± 5.3	0.848	−1.492	1.227
Hospital, day	11.9 ± 5.4	10.7 ± 61	0.139	−0.403	2.854
M.V duration, hours	140.9 ± 103.5	41.8 ± 47.8	0.002	−161.914	−36.266
Respiratory support, *n* (%)	90	114	≤0.001	—	—
Only invasive	2 (2.3)	27 (23.7)			
Only noninvasive	77 (85.5)	17 (14.9)			
Both	11 (12.2)	70 (61.4)			
Types of NIV, n			NA	—	—
Only F.M/N.C	8	15			
Only RBM	51	32			
Only CPAP mask	0	4			
Only BiPAP	0	5			
Combined	29	31			

GI: gastrointestinal, APACHE II: acute physiology and chronic health evaluation, SOFA: sequential organ failure assessment, ICU: intensive care unit, MV: mechanical ventilation, NIV: noninvasive ventilation, FM: face mask, NC: nasal cannula, RBM: reservoir bag mask, CPAP: continuous positive airway pressure, BiPAP: bilevel positive airway pressure, and NA: not applicable. APACHE II (adjusted OR, 1.21; 95% CI, 1.13–1.30) and mean SOFA of admission days (adjusted OR, 2.83; 95% CI, 2.09–3.83) scores were higher in the nonsurvived group significantly (*P* ≤ 0.001) (Table 2). In a cut-off point of 5 for SOFA and 13 for APACHE II, the area under the curve (AUC) was 89.5% and 73%, respectively (Figure 1).

**Table 2:** Odds ratio and 95% confidence intervals of APACHE II and mean SOFA scores and comorbidities of patients hospitalized for COVID-19 at intensive care unit.

Variable	Crude OR	*P* value	Adjusted OR	*P* value
APACHE II score	1.17 (1.1–1.2)	≤0.001	1.21 (1.13–1.30)	≤0.001
APACHE II group				
<13	Reference		Reference	
≥13	9.6 (4.9–18.9)	≤0.001	10.7 (5.0–22.9)	≤0.001
Mean SOFA score	2.7 (2.07–3.61)	≤0.001	2.83 (2.09–3.83)	≤0.001
Mean SOFA group				
<5	Reference		Reference	
≥5	33.7 (15.2–74.6)	≤0.001	32.4 (14.4–72.9)	≤0.001
Comorbidities				
No	Reference		Reference	
Any	2.7 (1.4–5.3)	0.004	2.1 (0.78–5.66)	0.14
Kidney diseases	4.23 (0.90–19.82)	0.067	7.12 (1.36–37.4)	0.020
Cardiovascular	0.87 (0.47–1.61)	0.66	0.71 (0.33–1.52)	0.384
Respiratory	4.73 (1.33–16.80)	0.016	8.56 (1.98–37.04)	0.004
Malignancy	2.52 (1.02–6.27)	0.04	4.35 (1.51–12.57)	0.007
Diabetes mellitus	1.07 (0.61–1.89)	0.80	1.61 (0.83–3.15	0.15
Hypertension	0.59 (0.34–1.05)	0.072	0.59 (0.29–1.25)	0.16

OR: odds ratio, APACHE II: acute physiology and chronic health evaluation, and SOFA: sequential organ failure assessment.

**Table 3:** Based on organic complications in survive and nonsurvive patients.

Variable	Survived *n* = 90	Nonsurvived *n* = 114	*P* value
Kidney, *n* (%)	20 (22.2)	87 (76.3)	≤0.001
AKI	14 (15.6)	73 (64)	≤0.001
Uremia	20 (22.2)	82 (71.9)	≤0.001
Oliguria	0	9 (7.9)	NA
Anuria	0	5 (4.4)	NA
Dialysis	1 (5)	15 (17.2)	0.001
Liver, *n* (%)	26 (28.9)	41 (36)	0.298
↑ Enzyme	23 (25.6)	25 (21.9)	0.619
Bilirubinemia	17 (18.9)	28 (24.6)	0.396
Coagulopathy	2 (2.2)	28 (24.6)	≤0.001
Hematologic, *n* (%)	78 (86.7)	104 (91.2)	0.365
Thrombocytopenia	6 (6.7)	24 (21.1)	≤0.001
Leukocytosis	36 (40)	66 (57.9)	0.016
Lymphopenia	69 (76.6)	103 (90.4)	0.017
Anemia	18 (20)	40 (35.1)	0.020
Heart, *n* (%)	3 (3.3)	70 (61.4)	≤0.001
↓ EF ≤40%	3 (3.3)	4 (3.5)	0.629
Vasopressor	1 (1.1)	70 (61.4)	≤0.001
Inotrope	1 (1.1)	13 (11.4)	0.001
Neurologic, *n* (%)	2 (2.2)	15 (13.2)	0.005
Loss of consciousness	0	10 (8.8)	NA
Seizure	0	0	NA
Lethargy	0	2 (1.8)	NA
Delirium	2 (2.2)	5 (4.9)	0.468
Paresthesia/plegia	0	1 (0.9)	NA
GI, *n* (%)	8 (44.4)	3 (50)	0.063
Bleeding	4 (22.2)	1 (16.6)	0.172
Diarrhea	4 (22.2)	2 (33.3)	0.409
Melena	2 (11.1)	0	NA
Respiratory, *n* (%)	2 (66.6)	23 (45.1)	≤0.001
Hemoptysis	0	6 (11.7)	NA
Pneumothorax	0	10 (19.6)	NA
Emphysema	0	5 (9.8)	NA
Effusion	1 (33.4)	7 (13.7)	0.080

AKI: acute kidney injury, EF: ejection fraction, GI: gastrointestinal, and NA: not applicable.

**Table 4:** Comparison of laboratory findings between survived and nonsurvived patients.

^*∗*^Variable	Survived mean ± SD	Nonsurvived mean ± SD	*P* value	95% CI	
				Lower	Upper
WBC	8971.3 ± 4564.6	12091.1 ± 5240.9	≤0.001	−4497.313	−1742.337
Neutrophil	78.4 ± 6.9	86.6 ± 7.6	≤0.001	−10.256	−6.276
Lymphocyte	15.7 ± 6.5	9 ± 5.5	≤0.001	5	8.329
Platelet	303261.3 ± 128630.4	232488 ± 117520.3	≤0.001	36702.921	104843.534
Hb	12.2 ± 1.7	11.6 ± 1.8	0.013	0.132	1.143
ESR	78.34 ± 23.8	76.50 ± 29.6	0.703	−7.674	11.336
CRP	77.1 ± 52.9	149.2 ± 86.1	≤0.001	−94.866	−49.363
Lactate	34.82 ± 9.7	34.50 ± 15.8	0.963	−14.115	14.764
BS	141.19 ± 44.2	176.78 ± 73.5	≤0.001	−53.676	−17.495
Urea	46.65 ± 24.9	81.11 ± 41.2	≤0.001	−44.244	−24.682
Creatinine	1.12 ± 0.7	1.65 ± 0.9	≤0.001	−0.765	−0.293
ALT	49.68 ± 33.4	95.49 ± 182.9	0.030	−87.026	−4.594
AST	44.94 ± 34.4	124.26 ± 354.1	0.045	−56.797	−1.832
Alk-P	200.61 ± 113.9	280.17 ± 229.9	0.006	−136.228	−22.893
Bili-T	1.46 ± 0.9	2.35 ± 2.2	0.002	−1.456	−0.332
Bili-D	0.40 ± 0.2	1.03 ± 1.3	≤0.001	−0.923	−0.336
Albumin	3.21 ± 0.3	2.70 ± 0.5	≤0.001	0.285	0.717
LDH	593.52 ± 159.4	865.03 ± 377.2	≤0.001	−382.606	−160.398
CPK	311.41 ± 1104.9	250.64 ± 292.4	0.702	−253.617	375.155
Pro-BNP	1577.18 ± 2670.3	1828.57 ± 1827.01	0.654	−1365.158	862.373
D-dimer	1797.69 ± 1222.7	4697.44 ± 5945.9	0.004	−4846.196	−953.316
Ca	8.15 ± 0.3	7.87 ± 0.5	≤0.001	0.166	0.453
PCT	2.38 ± 3.5	7.26 ± 19.7	0.266	−13.565	3.816
pH	7.42 ± 0.04	7.38 ± 0.1	≤0.001	0.022	0.059
PO_2_	73.52 ± 16.7	79.78 ± 26.3	0.120	−14.165	1.654
PCO_2_	37.2 ± 5.5	40.1 ± 10.1	0.021	−5.106	−0.426
HCO_3_	24.31 ± 3.6	23.76 ± 4.8	0.369	−0.657	1.757
BE	1.05 ± 3.5	−1.02 ± 4.5	≤0.001	0.946	3.226
FiO_2_	61.1 ± 10.7	80.6 ± 15.1	≤0.001	−23.335	−15.594
SPO_2_	92.8 ± 2.1	89.7 ± 6.3	≤0.001	1.727	4.454

^*∗*^Variables are the mean value duration of admission days. WBC: white blood cells, Hb: hemoglobin, ESR: erythrocyte sedimentation rate, CRP: C-reactive protein, BS: blood sugar, ALT: Alanine aminotransferase test, AST: aspartate aminotransferase test, Alk-P: alkaline phosphate, Bili-T: total bilirubin, Bili-D: direct bilirubin, LDH: lactic acid dehydrogenase, CPK: creatine phosphokinase, Pro-BNP: pro-brain natriuretic peptide, Ca: calcium, PCT: procalcitonin, PO_2_: partial pressure of oxygen, PCO_2_: partial pressure of carbon dioxide, BE: base excess, FiO_2_: fraction of inspired oxygen, and SPO_2_, peripheral capillary oxygen saturation.

## Data Availability

The datasets used and/or analyzed during the current study are available from the corresponding author on reasonable request.

## Abbreviations

APACHE II: Acute physiology and chronic health evaluation
SOFA: Sequential organ failure assessment
ICU: Intensive care unit
MuLBSTA: Multilobular infiltration, hypo-lymphocytosis, bacterial coinfection, smoking history, hypertension, and age
CURB-65: Confusion, urea, respiratory rate, blood pressure, age ≥65
WIC: Charlton's weighted index of comorbidities
ROC: Receiver operating characteristic
AUC: Area under the curve
CPAP: Continuous positive airway pressure
BiPAP: Bilevel positive airway pressure
COPD: Chronic obstructive pulmonary disease
ARDS: Acute respiratory distress syndrome.
